# A Re-Exploration of *our* Unconscious: What We Have Come To Unmask; What Still Lies Beneath

**DOI:** 10.1007/s12124-025-09935-2

**Published:** 2025-10-23

**Authors:** Myron Tsikandilakis, Persefoni Bali, Victοria-Maria Pasachidou, Romina Leonor Toranzos, Konrad Szczesniak, Pierre-Alexis Mével, Christopher Madan, Alison Milbank

**Affiliations:** 1 School of Psychology, University of Nottingham, Nottingham, UK; 2 Medical School, Faculty of Medicine and Health Sciences, University of Nottingham, Nottingham, UK; 3 School of Cultures, Languages and Area Studies, University of Nottingham, Nottingham, UK; 4 School of Science and Technology, Nottingham Trent University, Nottingham, UK; 5 Departamento de Letras Estrangeiras Modernas, Universidade Estadual de Londrina, Londrina, Brazil; 6 Institute of Linguistics, University of Silesia, Katowice, Poland; 7 Institute for Foreign Languages, Palacký University, Olomouc, Czechia; 8 Department of Philosophy, University of Nottingham, Nottingham, UK

**Keywords:** Unconscious, History, Theory, Method, Applications, Practice

## Abstract

In this manuscript, concepts, issues and resolutions that call for conscious awareness in research into the unconscious are revisited and reviewed. Historical episodes and episodes of controversial experimentation that are formative rallying points for understanding contemporary attitudes to the unconscious in psychological science, and the impact of historical controversy to contemporary polarisation, are meticulously discussed. The theoretical debates, methodological inquiries and contributing resolutions that have stemmed from these controversies are cited and discoursed. Replicated empirical illustrations, that show how purportedly established and well-known biases persist, and previously unaddressed methodological biases occur, and how we may resolve them, are experimentally demonstrated. We show that resolutions are still pending further advances within the – frequently overlooked – limitations of our current scientific paradigm. As a seminal communication, stemming from engaging with these themes, concepts, issues and resolutions, the contextual importance and research value of a *reminding*, and combined scholarly-theoretical and applied-empirical conscious understanding of unconscious research is carefully emphasised and accentuated.

## Rediscovering Antiquity

In a previous manuscript (Tsikandilakis et al., 2019a), the introductory section began with a mention of the earliest known reference to the unconscious in written language [τό ασυνείδητον] (see also Robinson, 2019). This was suggested to have been made by Plato in the Socratic Dialogues sometime approximately in the 3rd century B.C.E. (Reber, 2016). This mention was made in a purported discussion between Socrates and his friend Meno. During the discussion, Socrates questioned one of Meno’s uneducated servants. To Meno’s surprise, Socrates used Euclidean deductions to show to Meno that his servant was unconsciously using the Pythagorean theorem in his routes for completing his everyday tasks (Plato, 380–385 B.C.E./2009; pp. 70–86; see also Bronstein & Schwab, 2019).

Further study of the history of the unconscious led to discovering an earlier albeit fragmented mention of the unconscious in written language. The latter was made approximately in the 5th century B.C.E. by Heraclitus. In the few fragments that remain of his work, a particular passage reads as such: “…like people who dream without consciousness about wild and unreasonable events, some people live their waking lives without philosophical awareness and, therefore, unconscious of themselves and the world” (Heraclitus, 511–521 B.C.E./2003; see also Rankin, 1995; pp. 75–91).

The philosophical distance between the Heraclitan and Platonic concepts of the unconscious is significant. Their theses reflect the passage of ancient Greek philosophy from a Dionysian to an Apollonian era. The Dionysian era was an era of practical and applied philosophical awareness. The Apollonian era was an era of dialectical philosophy that was reserved for educated individuals (for a comprehensive review, see Madison, 1962).

Heraclitus proposed that being unconscious is a waking slumber characterised by lack of applied philosophical awareness, and that philosophical awareness is available to every thinking individual. Plato, on the other hand, advocated that the unconscious is part of a transcendental cosmos of archetypical ideas. He suggested that the majority of people cannot partake in and can only experience glimpses of that cosmos. He provocatively claimed that only the philosopher is privy to conscious access and awareness to this cosmos (Clark, 2010).

More than 2500 years since the advent of these concepts, the notion of the unconscious is still philosophically and scientifically debated, quite surprisingly in variations of these ancient models and conceptualisations (see Albarrak et al., 2021; Amd & Passarelli, 2020; Bargh & Morsella, 2008; Haggard et al., 2019; Ebbinghaus, 1908; Elgendi et al., 2018; Erdelyi, 2004; Kahn, 1943; Kihlstrom, 2018; Newell, & Shanks, 2014; Sandberg et al., 2022; Schiff, 1961; Szcześniak, 2024; Searle, 1989; Song et al., 2019; Soto et al., 2019). An important question, therefore, is: “Why?”.

### “Ghosts of Future Past” and Near-Contemporary Division Bells

To answer “Why?”, we have to return closer to our own time to a very controversial era in psychology. We have to begin our journey to an “appropriate” explanation with a provocative, and possibly even “inappropriate”, Derridean hauntology: It might not be a euphemism to submit that very many contemporary psychologists have read about Sigmund Freud; far fewer have read Sigmund Freud (see Westen, 1998a). Perhaps, by merely mentioning Freud in the beginning of this section, we might have already alarmed certain readers to a discarding internal monological dissent: “Not him!” or “Him again?” (see Western, 1998b). Nevertheless, the better questions towards the explanation we promised, and for the benefit of the better part of our readership (see Dawes, 2014) – as provocatively as this argument can be phrased – should be: “How did he, and whether and how does he continue to influence contemporary psychology?”

To this end, it is worth mentioning that some scholars believe that if there was no such thing as bad publicity, Freud might have been considered the most central individual to ever discourse the unconscious as a concept (see Westen, 1999). Nevertheless, for better or for worse, bad publicity can be unforgiving in psychological science (see particularly, Levine, 2000). Therefore, at present, we often forget that Freud was the first medically trained neurologist to address *madness* as a treatable medical disorder, and to provide a model for treating psychiatric illness involving a structured theory of the unconscious (see Leader, 2011).

On the other hand, and potentially to the defence of the contemporary anti-psychoanalyst, the Freudian theory of the unconscious involved provocative concepts (Freud, 1905/2005). It involved a bio-topographical arrangement of the human psyche (Freud, 1938/2003), and a theoretical-observational distinction of evolutionary-inherited phylogenetic mechanisms for denial and repression that could result in hysteria (for a comprehensive review, see Micale, 1989), such as ungovernable emotional trauma and persistent anti-social behavioural patterns (Freud, 1905/2013). It also included the proposition that the human condition involves a constitutional and involuntary apoptotic mechanism that is expressed via the controversial from an evolutionary survival value perspective workings of an instinct for death, or death instinct (Freud 1917/2005; for a comprehensive review, see Webster, 1995; see also Hoffman, 2004).

According to many scholars, Freud’s most provocative idea – and the one that has caused the most critical responses from several experimental psychologists – was that, like Plato and the Platonic notions of the world of archetypal ideas and the philosopher, he believed that access to the unconscious could only be achieved through psychoanalysis. For Freud, the unconscious was a physical space in the human mind that could only be explored by the privileged people who were privy to understanding the psychoanalytic method.

Freud’s provocative ideas were met with severe resistance by many theoretical and experimental psychologists (Burnham, 1967). His ideas – in their vast majority (Erdelyi, 1992) – were characterised first and foremost as unscientific. During the 1960’s and 1970’s particularly Karl Popper (Burnham, 1967), in a very extensive series of rather polemical publications (Popper, 1950, 1952, 1959, 1960), that reached their climax in his *Conjectures and Refutations* (1963), accused the Freudian notion of the unconscious of being irrefutable and unfalsifiable. These accusations were subsequently met by several psychoanalytic authors with claims that they reflected personal traumas relating to the criticizing authors (Grünbaum, 1979). These responses were in turn considered a confirmation of the original criticisms relating to the infallibility of the psychanalytic method (Frosh, 1989).

As a result, the psychoanalytic method was considered guilty by the majority – but not the entirety (see Bornstein, 2005) – of psychological empiricism for having *failed to become falsifiable*, and, along with psychoanalysis, the notion of the unconscious was also severely stigmatised (see Phillips, 2014). Quite inconveniently, at approximately the same period in history that the psychoanalytic method and psychological empiricism were divided, another battlefront for the unconscious emerged. Infamy, scepticism and controversy were stirred in 1957 when James Vicary claimed that, during random movies, unbeknown to the attending audiences, he presented very brief unconscious messages (i.e., 18 milliseconds; 1/55th of a second) in cinema theatres throughout the United States. These messages included statements such as “Drink Coca-Cola” and “Eat Pop-Corn”. He claimed that he was able to boost Coca-Cola and pop-corn sales in these cinema theatres by 18.1% and 57.5% respectively using unconscious messages. His claims caused scepticism and, eventually, under the threat of legal inquiry, and intense pressure by the scientific community to disclose his findings, James Vicary retracted. In 1962, James Vicary admitted that he had no research data to disclose (Rogers, 1992). He admitted that his claims were an elaborate advertising and publicity hoax (Nelson, 2008).

### Polarisation

For several experimental psychologists, this admission was conclusively stigmatising. It added insult to injury and was received as cause and occasion for a complete departure from the possibility of sober empirical research on the unconscious. Several other psychologists adopted the view that completely rejecting research into the unconscious due to confrontational controversies was a very partial reading of the empirical history of the unconscious in psychological science. Several psychologists believed that from Ebbinghaus (1908), and MacLean (1949) to, eventually, Macmillan and Creelman (2004), a robust science relating to the unconscious had existed and should exist (for a comprehensive review, see Kihlstrom, 1990).

The divide created confrontational polarities. For part of the psychological community, research on the unconscious could provide significant insights into human cognition. For others, research on the unconscious was unreliable, uncredible, it had very little to offer to psychological science and should be avoided at all costs from dignified experimental research (see Shanks et al., 2021; Stafford, 2014; Talvitie, 2018; but see also Bar & Biederman, 1998; Elgendi et al., 2018; Merikle & Cheesman, 1987; Pessoa & Adolphs, 2010).

### Reconciliations

**Fig. 1:**
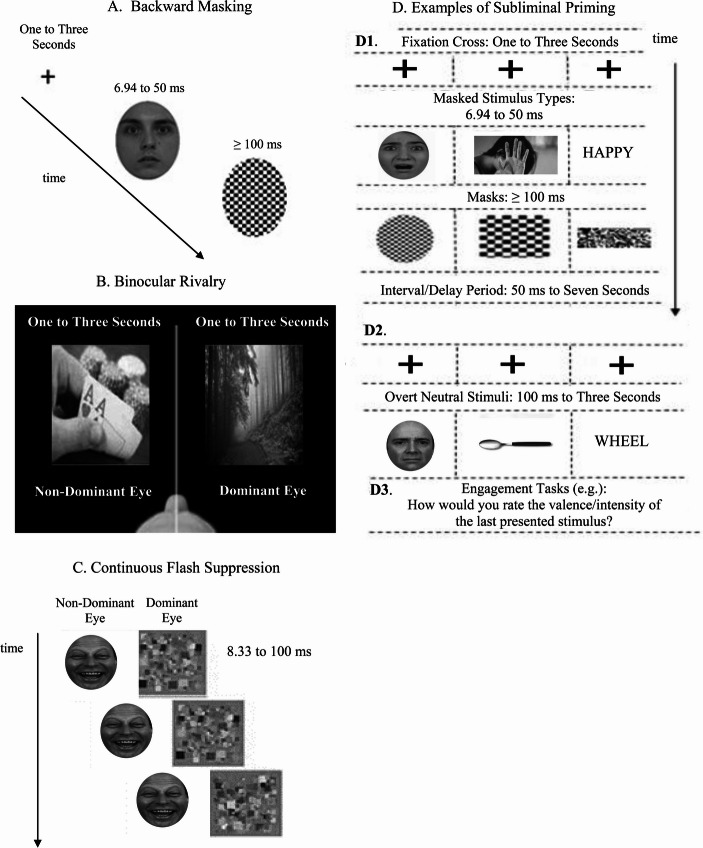
Different methods for visual. In (**A**) Backward Masking, in (**B**) Binocular Rivalry and in (**C**) Continuous Flash Suppression (Blake & O’Shea, 2009; Stein et al., 2020; Knotts et al., 2018). These methods are suggested to stem from similar principles and to share common objectives (Faivre et al., 2012). In these methods, a target elicitor with typically semantic or emotional characteristics is presented. This target is usually presented for brief durations (e.g., 6.94 to 100 ms; Breitmeyer, 2015) and it is either subsequently or simultaneously masked with a neutral image or images that are suggested to render the target imperceptible (Kim et al., 2010). The effects of the masked target are assessed using self-reports and physiological assessments (Brooks et al., 2012; van der Ploeg et al., 2017; Meneguzzo et al., 2014). In the case of unconscious priming, the effects of the target are assessed in relation to a subsequently presented typically neutral overt stimulus, to explore whether the target unconsciously influenced participant responses to the overt stimulus (Van den Bussche et al., 2009)

Eventually, history showed that middle ground was preferable to extremities (Hassin et al., 2004; Kihlstrom et al., 1992; Scherer, 2005; Shevrin & Dickman, 1980; Westen, 1999). Subjects, such as the psychophysics of signal detection, the engineering of psychophysiological assessments for exploring unconscious emotional responses, and empirical visual suppression methodological paradigms for the assessment of unconscious emotional responses, could not be reduced to pseudoscience (Siegler, 2000; Tallis, 2002; Winkielman & Berridge, 2004; see also Fig. 1). Furthermore, the general public persisted in showing interest in this subject (Elgendi et al., 2018; Song et al., 2019). As a result, research and publications exploring unconscious processes continued (Albarrak et al., 2021; Haggard et al., 2019; Soto et al., 2019). Research and publications discrediting the exploration of unconscious processes continued as well (Scherer, 2005; Stafford, 2014; Stein, 2019).

### “Empirical Evidence” of Reparation

From these theses and antitheses, a contributing synthesis emerged. Many psychologists believed that the topic of polemical polarization were not the inevitable paradigm-schisms and intentional distancing among experimental psychology, and the psychoanalytic method and falsified psychological findings respectively. They believed that the true cause of polemical polarization was an argumentative within-experimental-psychology division of empirical perspectives (Ekstrom, 2004). Some psychologists – originating frequently from a cognitive-affective neuroscience background (Bargh, 2011) – believed in unconscious processes; others – originating frequently from a more conservative cognitive-behavioural background (Greenwald & Banaji, 2017) – did not. The aforementioned controversies simply brought these predilections to explicit confrontation (Kihlstrom et al., 2000).

As a result, a moderating group of theoretical and experimental psychologists emerged (see for example, Norman, 2010). Potentially, their most insightful contribution was that they believed that whether unconscious processes occur, or not, bore significant theoretical and applied value for psychological science (see Kihlstrom, 2018). They believed that the real question was not which side or tradition one was inclined to choose between whether cognition, behaviour and emotion can function automatically and involuntarily, or in response to invisible elicitors, and despite our own volition and understanding (see Wang & Minor, 2008); it was simply: “Can they?” (see Bargh & Hassin, 2021). A science of the unconscious was necessary. It was required to understand how human perception, attention, emotion, and behaviour operate, and, fatefully, therefore, whether unconscious processes occurred, or not, could change psychological science as a whole (see Shevrin & Dickman, 1980).

Towards this end, instead of discarding research on the unconscious as a whole, and proverbially “throwing away the baby with the bathwater”, research on the unconscious was assessed for the implementation of methodological innovations and advances (Breitmeyer & Ogmen, 2000; Strick et al., 2011; Wang et al., 2017). This movement involved a significant shortcoming: Experimental psychologists engaged considerably less with the conceptual *zeitgeist* of what the unconscious is because partialities concerning “what it is, or not?” were already the subject of the divisive debate. It also involved a significant advantage. Experimental psychology engaged with noticeably reduced polemical predilections to empirically answering a simple but significant question: “Do unconscious processes occur?”.

This was not yet adequate for conferring universally accepted conceptual insights as to the definition of what the unconscious is, but it was sufficient to set a consensual methodological paradigm: Unconscious processing was initially empirically defined as emotional or behavioural responses to imperceptible elicitors (see Erdelyi, 2004). This empirical definition was a Voltaire’s Candide solution to the conceptual exploration of the cognates of conscious and unconscious processes. Nevertheless, it set a course for empirical researchers to attaining outcomes based on applied methodological unanimity (Bargh, 2017).

Proverbially, therefore, *limping* from a conceptual accord standpoint, but involving at-the-very-least methodological consensus as to its exploration, experimental research into the unconscious was promptly and firmly assessed as regards its validity (Meyen et al., 2022). This involved an exploration of the metrics (Macmillan & Creelman, 2004) and analyses (Faivre et al., 2012) applied for assessing the unconscious. It involved an assessment of the statistical and mathematical definitions for presenting stimuli that should be considered to have been presented unconsciously (Dienes, 2014, 2015, 2016), and involved advances in visual psychophysics related to visual suppression (Breitmeyer, 2015).

## A First Focal Point

Several potentially reliable methods for testing whether unconscious processes occurred had been proposed for researchers to explore how to implement advances and resolutions. These methods included continuous flash suppression (Blake & O’Shea, 2009), binocular rivalry (Knotts et al., 2018) and backward masking (Stein et al., 2020; see Fig. 1). Topical research focused, largely, on backward masking, and, specifically, backward masking using faces (Breitmeyer & Ogmen, 2000). This occurred because backward masking was arguably easier to implement compared to its alternatives (Breitmeyer, 2015 Breitmeyer & Hesselmann, 2019). It did not require portable or external devices, such as 3D red-cyan glasses or ocular aids, like binocular rivalry and continuous flash suppression did (Hermens et al., 2008). These advantages – or, according to certain researchers, methodological conveniences (see Tsuchiya et al., 2006) – were strongly reflected in relevant meta-analytic research. Therefore, backward masking was and is the most widely reported method for visual suppression in meta-analyses on research relating to the unconscious for the past fifteen years and until this very day (k _studies with backward masking/overall reported studies_ = 126/187; see Brooks et al., 2012; pp. 2964–2965 Bussche et al., 2009; pp. 462–463; Gambarota et al., 2022; pp. 7–9; Mertens & Engelhard, 2020; pp. 259–261;Van den; Meneguzzo et al., 2014; pp. 10–11; van der Ploeg et al., 2017; pp. 141–143).

### Backward Masking

Backward masking – that was perhaps, surprisingly so, quite akin to the method that Vicary used to fabricate his research (see Pratkanis, 1992) – referred to the presentation of a brief target stimulus, such as typically, but not exclusively, an emotional face (for a dedicated review, see Axelrod et al., 2015). During backward masking the target stimulus was presented for a brief duration, such as 16.67 ms (i.e., a single frame of a 60 Hz monitor). The target stimulus was subsequently followed by a noise image presented typically for 100 to 150 ms (Kim et al., 2010). The aim of the noise image was to disallow conscious perception of the target stimulus. After the presentation, peripheral nervous system (PNS) psychophysiological assessments could be employed. These could involve subcutaneous sweating responses (known as skin conductance responses, or SCR). The latter were commonly measured as the highest peak in microsiemens (µS) during a three-second post-stimulus offset window when compared to a pre-stimulus onset baseline (Cacioppo et al., 2007; pp. 159–177). These could also involve heart-rate (HR) responses. These were commonly measured as the highest peak in beats-per-minute (bpm) during a five-second post-stimulus offset window when compared to a pre-stimulus onset baseline (Cacioppo et al., 2007; pp. 182–205).

The psychophysiological assessments could also involve central nervous system (CNS) assessment methods, such as fNIRS, EEG and fMRI (for a thorough review, see Brooks et al., 2012). Nevertheless, these assessment methods often suffered the disadvantages that they could be costly, time-consuming and resource-demanding, often resulting in small sample sizes (Cremens et al., 2017). Many researchers raised critical concerns as regards whether the studies that provided outcomes using these assessments involved false-positive (Type-I error) or false-negative (Type-II error) results (see Bossier et al., 2020). The evaluation of the outcome validity of these methods became the subject of disagreeing psychological perspectives (for thorough reviews on this subject, see Feng et al., 2022; Elliot et al., 2020; van der Ploeg et al., 2017).

In addition, participant self-reports (Bond et al., 2021; Haralabopoulos et al., 2020; Leong et al., 2024; Yu et al., 2022; Leveridge et al., 2024; Mevel et al., 2025), typically, but not exclusively, for valence and intensity were often recorded to assess the experience of the participants during the presentation of backward masked stimuli (Macknik & Martinez-Conde, 2007). The aim of these assessments was to explore whether a researcher could provide evidence for physiological and/or behavioural responses to stimuli that did not provide evidence for conscious perception, or awareness (see Weinberger & Stoycheva, 2019; see also Fig. 1).

## Static Methods and Methodological Change

With backward masking as a rallying point and a general consensus concerning the assessment methods for reporting unconscious responses, two problematic and extensively debated issues were soon prioritised for emendations (Wiens, 2006). These issues were the use of hit rates as a metric for measuring perception, and null hypothesis significance testing (NHST) as a statistical method for determining whether a presentation was truly unconscious (see Enns & Di Lollo, 2000; Kim & Blake, 2005).

Hit rates, in the current research area, were defined as the percentage of correct answers for detecting visually suppressed stimuli. The issue with hit rates was that they were affected by response biases. For example, some participants would respond conservatively, that is, they would withhold a response for having seen a stimulus unless they were absolutely certain that a stimulus was presented. Others would respond liberally, that is, they would respond having seen a stimulus even if they were quite uncertain whether a stimulus was presented (Stanislaw & Todorov, 1999).

NHST is a statistical method that, employing, in this area, an analysis called a one-sample t-test, could allow a researcher to conduct a comparison between the participants’ detection responses and mere-guess chance-level detection responses (e.g., 50%), such as responses to imperceptible stimuli or, in more colloquial terms, responses that we would expect from a blind individual. This method could provide evidence for whether a researcher could obtain significant or non-significant results, meaning whether they were able or whether they were not able to reject the null hypothesis that the participants’ detection performance was not statistically different to chance. In case of non-significant results, such as failing to reject the null, the outcomes of this analysis were erroneously interpreted to show significance for proximity, such as evidence for the null (Dienes, 2015, 2016).

### A Conceptual Example

As a conceptual illustration, let us assume that we are presenting, using backward masking, fearful, sad and neutral faces for 16.67 ms using a 100 ms mask. After every trial we ask “Did you see a face? (Y/N)”. When using hit rates, we cannot control for the possibility that the participants can reply using either conservative response strategies such as replying that they saw a face only when certain beyond a shadow of a doubt that a face was presented. We also cannot control for liberal response strategies and criteria, such as participants replying that they saw a face even when quite uncertain that a face was presented. Many researchers might not feel moved by these possibilities and proceed to test the resulting data. They could use a one-sample t-test and, for the purposes of this hypothetical scenario, report that participants’ detection performance was not significantly different to chance (*p* <.05).

That would mean that using a biased metric (hit rates), we have failed to reject the hypothesis that the participants’ detection performance was not significantly different to chance. We could claim that we have evidence for the presentation of unconscious faces (see Brooks et al., 2012; Meneguzzo et al., 2014; van der Ploeg et al., 2017). In reality, we employed a biased metric for perception which we used to apply a statistical analysis. In this statistical analysis, we misinterpreted failing to reject the null hypothesis, that there are no significant differences between the participants’ performance and chance, with providing direct evidence that confirm the null hypothesis, that the participants’ performance and chance were statistically proximate (see Dienes, 2016).

### A Practical Illustration, Part I: “To Seek”

**Fig. 2:**
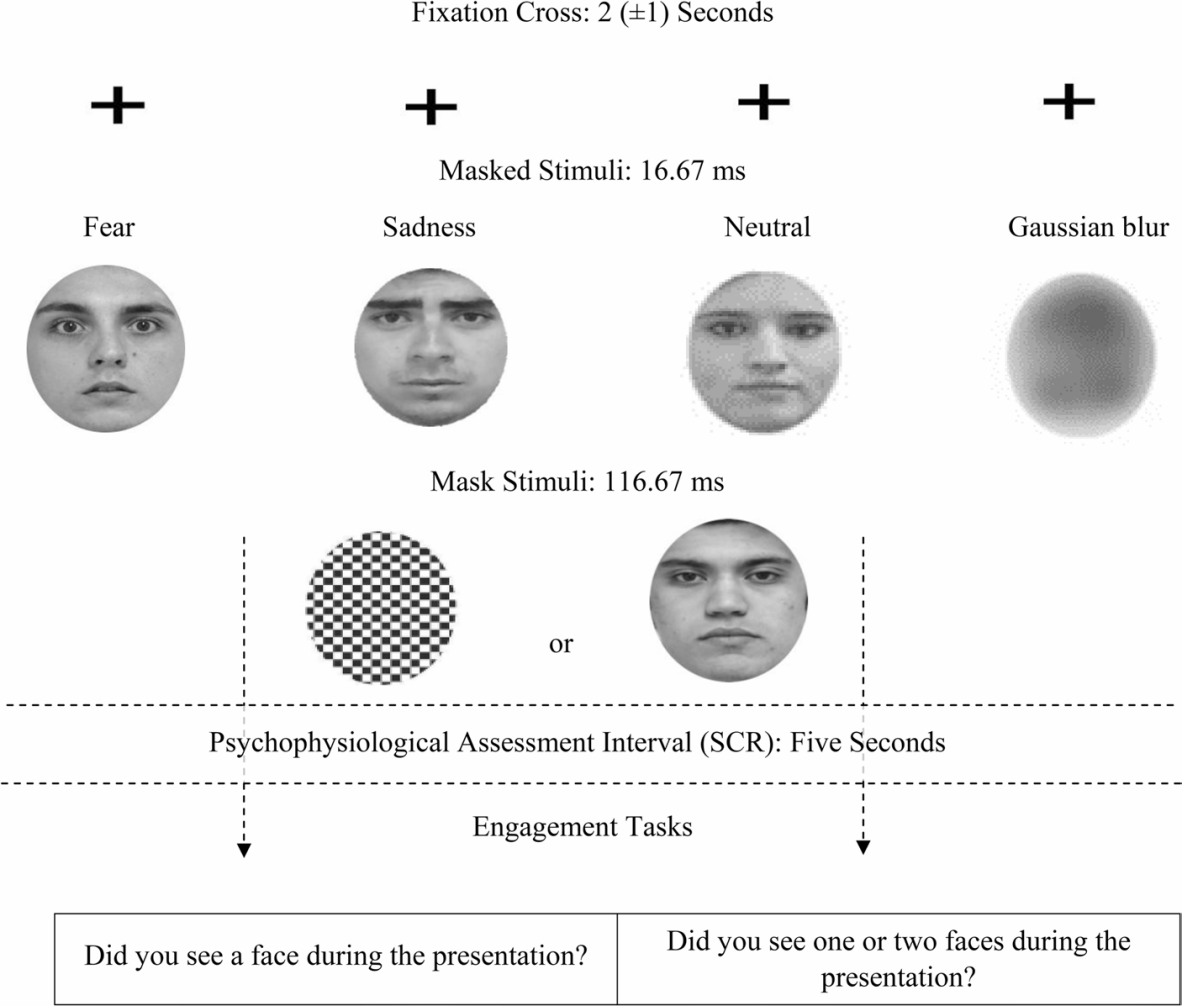
Experimental design. Pilot study one. An Illustration of a 2017 pilot study including fearful, sad and neutral faces, and an equal number of Gaussian blurs. The stimuli were presented for 16.67 ms (60 Hz monitor) and were masked either by a black and white pattern in one session or a neutral face in one session. Skin-conductance responses were measured from the left hand (index/first and middle/second fingers) of each participant using disposable Ag/AgCl gelled electrodes. The signals were received by a BIOPAC System, EDA100C in units of microsiemens (µS) and recorded in AcqKnowledge (Braithwaite et al., 2013). The presence of a phasic skin conductance response was defined as an unambiguous increase (0.01 µS) with respect to each pre-target SCR score occurring one to three seconds post-stimulus offset (Critchley, 2002; Williams et al., 2005). After the SCR assessment participants were asked whether they a face during the presentation (Y/N) for the pattern mask session or whether they saw one of two faces during the presentation for the neutral mask session (1/2)

As a practical illustration, in an unpublished pilot study that we conducted in 2017, we recruited a total of 120 undergraduate psychology students (69 female) (P _(1−β)_ ≥ 0.9; *p* ≤.05; η^2^_p_ = 0.06; f = 0.25). The mean of the population sample in years was 20.81 (SD = 1.07). We engaged the participants in two same-day experimental sessions. In each session, twenty fearful, sad and neutral faces (Gur et al., 2002), and sixty Gaussian blurs, created in MATLAB (see Fig. 2) were presented for 16.67 ms. In one session, the brief stimuli were backward masked with neutral faces for 116.67 ms. In one session, the brief stimuli were backward masked with black-and-white patterns for 116.67 ms. The order of the different-masked sessions was randomised. The participants were allowed a five-minute break between sessions. After each stimuli sequence a blank screen was presented for five seconds during which we measured peripheral nervous system arousal using SCR (Cacioppo et al., 2007; pp. 159–177). After the five-second interval, participants in the neutral-mask session were asked to decide how many stimuli were presented during the trial. Participants were asked to press “1” if they saw one face or “2” if the saw two faces, using the keyboard. Conversely, in the pattern-mask session, the participants were asked “Did you see a face?” (Y/N; for a topical overview and discussion on response-choice options, see van der Ploeg et al., 2017; see also Fig. 2).

The detection performance results for the neutral-mask session were overall 50.09% (SD = 1.29%). If we compare our results to chance using a one-sample t-test (50%) we report t (119) = 1.26; *p* =.211 with an effect size – that is very frequently not presented for this type of analysis because it requires manual calculations (see Rosnow & Rosenthal, 2003) – defined as $\:d=\frac{Sample\:Mean-\:Theoretical\:Comparison\:Mean}{SD\:(n-1\:)}=.07$. Our results showed that detection performance was not significantly different to chance.

SCR scores showed that there were significant differences between Types of Emotion (Fear vs. Sadness vs. Neutral) (F (1.77, 210.43) = 251.23; *p* <.001; η^2^_p_ = 68; ε = 0.88; Greenhouse-Geisser corrected). Further Bonferroni-corrected comparisons revealed that fearful faces (M = 0.125; SD = 0.035) led to higher SCR compared to sad (M = 0.065; SD = 0.021; *p* <.001; d = 2.23) and neutral faces (M = 0.052; SD = 0.022; *p* <.001; d = 2.71). Our study showed outcomes for higher arousal as measured by SCR (µS) for fearful compared to sad and neutral faces. These results could be interpreted to support that fearful faces elicited unconscious emotional responses, but they should not (see Tsikandilakis & Chapman, 2018; Tsikandilakis et al., 2018, 2019a, b, c).

As mentioned above, hit rates are subject to conservative and liberal response strategies and criteria. Therefore, we require a more reliable metric for the assessment of detection performance. Instead of hit rates, we can use receiver operating characteristics, such as sensitivity index A. We propose sensitivity index A based on advantages that A has compared to d’, A’ and A’’. For example, compared to d’, A is a nonparametric index and does not include any assumptions concerning the shape of the underlying noise-to-signal distribution. Sensitivity index A can provide an index for zero values, such as zero hits or miss responses, and includes diagonal Euclidean corrections to the A’ and A’’ metrics for scores that lie in the upper left quadrant of the ROC curve (i.e., False Alarms Rate ≤ 0.5 & Hit Rate ≥ 0.5; see Zhang & Mueller, 2005; pp. 204–207). The absence of this sensitivity metric in relevant research is arguably (see Swets, 2014) due to the mathematically challenging exposition of the algorithmic functions in the original publication (Zhang & Mueller, 2005; pp. 203–209). Nevertheless, currently easy-to-use calculators for sensitivity index A have been made available for use by the current and other research groups (see Macmillan & Creelman, 2004; see https://sites.google.com/a/mtu.edu/whynotaprime/; see also https://osf.io/x5jpc/).

Sensitivity index A can provide a ratio between hits, responding that a masked face was presented when a masked face was presented, and false alarms, responding that a masked face was presented when a masked face was not presented. This approach, by taking into account “error” as a contributing variable to the assessment of perceptual performance, is significantly less affected by conservative or liberal strategies and can provide a reliable metric for the perception of visually suppressed stimuli.

Moreover, we already discussed that NHST cannot provide us with evidence that perception is at-chance. This method can only provide us with evidence that we can reject the null hypothesis that perception was not different to chance. Bayesian analysis can provide direct evidence for chance-level perception. Bayesian analysis requires a lower (LB) and an upper bound (UB). These bounds are called credible intervals. They stand for the lowest and highest values within which we can define a meaningful range for exploring whether the participants’ perception was proximate to chance-level perception (e.g., A _chance−level_ = 0.5; Lower Bound (LB) = 0.4 or 0.45; Upper Bound (UP) = 0.6 or 0.55; see Tsikandilakis et al., 2020a). The credible intervals can be based on previous research findings, or a theoretically-driven rationale (Schönbrodt & Wagenmakers, 2018) or based on pre-determined study-specific expectancies, such as end-user requirement characteristics, or study-specific minimum effect sizes of interest (e.g., η^2^_p_ ≥ 0.01 or Cohen’s d ≥ 0.2; for a dedicated review, see Dienes et al., 2018).

Bayesian inference requires the standard error of the population sample, and a simple deduction of the sample mean from chance to provide a Bayes Factor (BF; see Dienes, 2014). The BF shows at a BF < 0.33 direct evidence for the likelihood of the data being observed if the null hypothesis is true (i.e., chance-level detection performance), at 0.33 < BF < 3 that the results are inconclusive, and at BF > 3 evidence for the likelihood of the data being observed under the alternate hypothesis, that participants’ detection performance was substantially different to chance (Dienes, 2015; for the Bayesian metrics presented above see http://www.lifesci.sussex.ac.uk/home/Zoltan_Dienes/inference/Bayes.htm).

When applying sensitivity metric A in our data, we report that overall the detection performance in the neutral-mask session of our pilot study was 0.61 (SD = 0.03). We could calculate the standard error using any standard interactive software (e.g., SPSS, STATA, MEDCALC) but for transparency reasons we can calculate it here as $\:Standard\:Error=\frac{Standard\:Deviation}{sqrt\:\left(n\right)}=\frac{.03}{10.95}=\:.002$. A Bayesian analysis with meaningful credible interval for chance-level performance for our participants could be between a lower bound of 0.5 (absolute chance) and upper bounds of 0.55 (conservative interval; i.e., Cohen’s d ≥ 0.2) or 0.6 (liberal interval; i.e., Cohen’s d ≥ 0.5). Our mean difference between chance (0.5) and our population (0.61) is 0.11. Using these values, we can fully populate a Bayesian function (see Dienes, 2016; for available code and software see https://osf.io/rm7qe/).

Using sensitivity index A and Bayesian analysis, we get, for both our conservative and liberal upper bounds, a BF = +∞. This is signifying that the likelihood of the data being observed under the alternative hypothesis is almost certain. Therefore, we can carefully submit, and have showed, that the reported skin conductance outcomes of our pilot were not physiological correlates to mere-guess/chance-level detection responses (i.e., imperceptible faces; see particularly, Macmillan & Creelman, 2004; pp. 3–5 & 9–12).

### A Practical Illustration, Part II: “To Hide”

In the same experiment, the participants were presented in randomised order with masked fearful, sad and neutral faces, and Gaussian blurs with either neutral-face masks or pattern masks. In the neutral-face mask session, participants reported A = 0.69 (SD = 0.04) for masked fearful faces, A = 0.61 (SD = 0.03) for sad faces and A = 0.52 (SD = 0.02) for neutral faces, aggregating to A = 0.61 (SD = 0.03) for overall detection performance. If we apply the same metrics in the pattern-mask session, participants reported A = 0.56 (SD = 0.02) for fearful faces, A = 0.55 (SD = 0.03) for sad faces and A = 0.55 (SD = 0.04) for neutral faces, aggregating to A = 0.56 (SD = 0.03). If we input these detection performance scores in a repeated measures ANOVA with independent variables Types of Masking (Neutral-Face vs. Pattern) and Types of Emotion (Fearful vs. Sad vs. Neutral), we find significant differences between Types of Masking (F (1, 119) = 1195.28; *p* <.001; η^2^_p_ = 0.91; SE = 0.005; BF = +∞), Types of Emotions (F (1.5, 178.83) = 796.03; *p* <.001; η^2^_p_ = 0.87; ε = 0.92; Greenhouse-Geisser corrected; SE = 0.006; BF = +∞), and a significant interaction (F (1.47, 174.74) = 44.59; *p* <.001; η^2^_p_ = 0.27; ε = 0.91; Greenhouse-Geisser corrected; SE = 0.013; BF = +∞).

Further analysis shows that the neutral-mask session was significantly higher for overall detection performance compared to the pattern-mask session (*p* <.001; η^2^_p_ = 0.58; SE = 0.012; BF = +∞). Bonferroni-corrected comparisons show that neutral-masked fearful faces were higher than neutral-masked sad (*p* <.001; d = 2.26; SE = 0.002; BF = +∞) and neutral faces (*p* <.001; d = 5.38; SE = 0.001; BF = +∞), and pattern-masked fearful (*p* <.001; d = 4.11; SE = 0.002; BF = +∞), sad (*p* <.001; d = 3.96; SE = 0.001; BF = +∞), and neutral faces (*p* <.001; d = 3.5; SE = 0.001; BF = +∞). Neutral-masked sad faces were higher than neutral-masked neutral faces (*p* <.001; d = 3.53; SE = 0.001; BF = +∞), pattern-masked fearful (*p* <.001; d = 1.96; SE = 0.002; BF = +∞), sad (*p* <.001; d = 2; SE = 0.002; BF = +∞) and neutral faces (*p* <.001; d = 1.69; SE = 0.002; BF = +∞).

Until now, these results make the simple case that pattern masks are better than neutral-face masks for visually suppressing fearful, sad and neutral faces. This is a simple point elaborated thoroughly because it has important implications. Contrary to the bulk of our results, neutral-masked neutral faces were lower for detection performance than pattern-masked fearful (*p* <.001; d = − 2; SE = 0.001; BF = +∞), sad (*p* <.001; d = − 1; SE = 0.002; BF = +∞) and neutral faces (*p* <.001; d = − 0.95; SE = 0.002; BF = +∞). These effects are antithetical to the higher detection for the neutral-masked session compared to the patterned-masked session effects we consistently reported in our other results. They present us with a seemingly inexplicable effect. Nevertheless, if we look closely at Fig. 2, we can observe a very clear explanation for this effect: In the neutral-mask session, for neutral faces only, we masked neutral faces with neutral faces.

Detection performance was higher for fearful and sad faces compared to neutral-masked neutral faces because the presented trials involved masked-to-mask stimulus mismatch. In this experimental implementation, we tried to mask neutral and non-neutral faces with neutral faces. This resulted in the only occasion in our entire design of a BF of 0.7 that approximated evidence for the null that neutral-masked neutral faces were imperceptible to the participants. This effect can be particularly insidious in designs that compare one or more neutral-masked emotional faces to neutral-masked neutral faces (Balconi & Lucchiari, 2007)^1^. Therefore, as a conclusive result of this empirical illustration, we strongly suggest that the traditional, and frequently applied method of backward masking emotional and neutral faces with neutral faces should be avoided. Pattern masks are the better choice for masking emotional faces, and potentially emotional stimuli in general (see particularly, McCrackin et al., 2023; see Fig. 1)^2^.

## A Serious Issue with Resolutions

Before we proceed to more novel issues and resolutions in this area, it is worth considering the reasons for which we chose to conceptually and empirically scrutinise so firmly the above topics, that many researchers might dismiss as repetition and consider “common knowledge”. The reason is simple: They are not “common practice”. A very important issue with purportedly “common knowledge”, “easily-accessible” and “well-established” resolutions is that they are not applied. As an illustration of our thesis, we can return to the “Backward Masking (2.2)” section in the current manuscript. In that section, we cited the backward-masking studies included in the six most recent meta-analyses in this area.

In Van den Bussche and colleagues (2009; pp. 462–463), we were presented with twenty-three studies using backward masking. Three studies used signal-detection-theory metrics (d’) and no study used Bayesian analysis. In Brooks and colleagues (2012; pp 2964–2965), we are presented with a total of eleven studies that employed backward masking, three of which use signal-detection-theory metrics (d’), and none of which used Bayesian analysis. Similarly, in Meneguzzo and colleagues, (2014; pp. 10–11), we are presented with an additional nine studies that used backward masking, three of which used signal-detection metrics (d’) and none of which used Bayesian analysis. In van der Ploeg and colleagues (2017; pp. 141–143), we were presented with an impressive array of sixty-five studies, forty-seven of which employed backward masking, seven of which employed signal-detection metrics (d’ & A’) and none of which employed Bayesian analysis. To their credit, the specific authors are the only ones in the relevant list who raised the issue of the lack of using signal-detection-theory measures for assessing subliminal processing (see van der Ploeg et al., 2017; pp 0.149–150). In Mertens and Engelhard (2020; pp. 259–261), we were presented with thirty studies, twenty-one of which employed backward masking, four of which used signal-detection-theory measures (d’ & A’), and none of which used Bayesian analysis. Finally, in Gambarota and colleagues (2022; pp. 7–9), we were presented with an adjusted to Bayesian inference meta-analysis of a total of twenty-four studies, seventeen of which used backward masking, none of which used Bayesian analyses in their original reports, and four of which used signal-detection-theory measures (d’). All the aforementioned studies used neutral-faces, scenery or items masks (see Kim et al., 2010; see also Jimenez et al., 2025).

The available bibliography shows that between 2009 and 2022 a total of one-hundred-twenty-six studies using backward masking have been assessed by meta-analytic research. Twenty of these studies used d’ or A’ instead of hit rates, no study used Bayesian analyses, and all studies used neutral masks. Three of the key “purportedly established” issues and resolutions we discussed until now are almost provocatively missing from the vast majority of contemporary empirical psychological research. Our initiative and tenacity for engaging with these subjects is – as simply as we can make our case – because they are not included in the vast majority of contemporary empirical research (see Stanley, 2017).

## Beyond “The Purportedly Established”: the Psychophysics of Image Processing

**Fig. 3:**
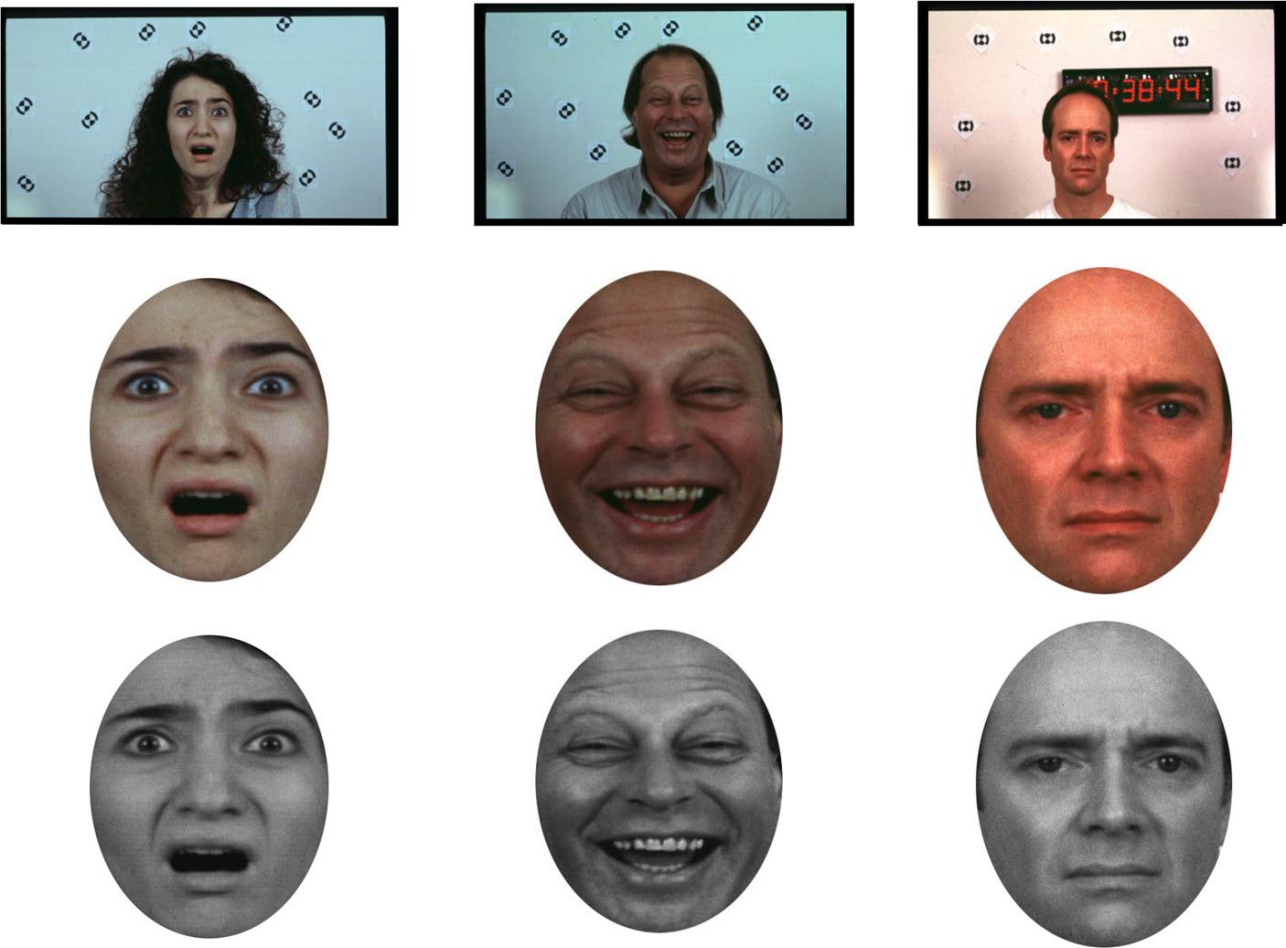
Processing emotional faces. Example of processing facial images from their native dataset to an experimental design relating to the unconscious. From left to right fearful, happy and sad expressions are presented. The processing of such expressions typically involves cropping into a fixed size oval shapes with pure white backgrounds. Then the images are subjected to grayscaling to remove between-stimuli colour contrast artifacts and presented as part of the experimental design (Brooks et al., 2012; Meneguzzo et al., 2014; van der Ploeg et al., 2017)

Subsequently, and forward from these “purportedly established issues”, we should be wise to examine more contemporary concerns related to the psychophysics of image processing (Willenbockel et al., 2010). For example, the typical processing of backwards masked emotional faces includes cropping, adjustments for interpupillary distance and grayscaling. These refer, respectively, to placing a face into a cropped circle, removing the image background, adjusting the positioning of the eyes at fixation within the cropping circle, and removing colour artifacts by using shades of grey for presenting emotional faces (see Gray et al., 2013; see Fig. 3).

**Fig. 4:**
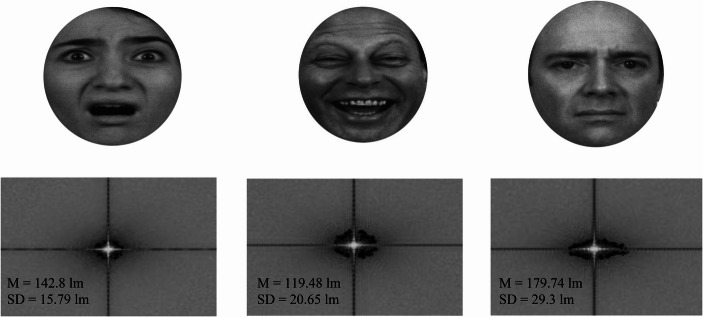
Discrete fourier transformations. Discrete Fourier Transformation of the images in Fig. 3. The mean and standard deviation of the images are presented in units of lumens. Instead of convolutional base filtering and multiplication by each pixel this methods allows us to map and calculate the low frequency components in the centre of the image, i.e. luminosity, and the high frequency components, structure and edge in the peripheral/bilateral regions of the plot. In this case, the luminosity scores of the three images provide simple z-score evidence for being ≥ ± 2/3 standard deviations apart. This supports our thesis that applying grayscale corrections to these facial images maintained the colour contrast of the original images

**Fig. 5:**
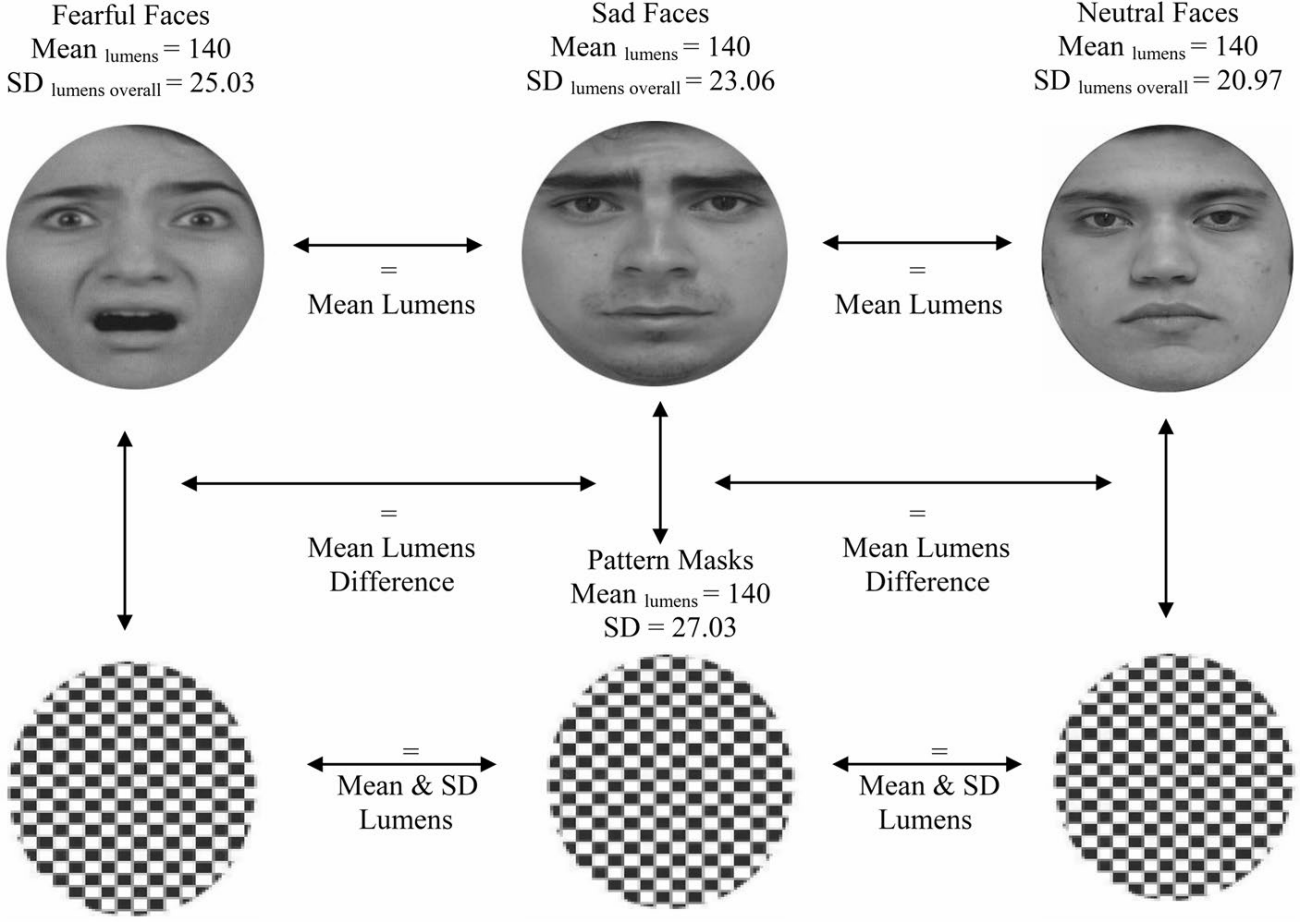
Equivalence of contrast. Equivalence of contrast between masked and masking stimuli mean lumens after Fourier and SHINE, MATLAB processing of their psychophysics characteristics. Face stimuli, mask stimuli and masked-facial to masking-pattern difference combinations of stimuli were presented using mid-lumens adjustments (M = 140) resulting in a reduction of soft component characteristics differences in the presentation of masked faces and masking patterns

**Fig. 6:**
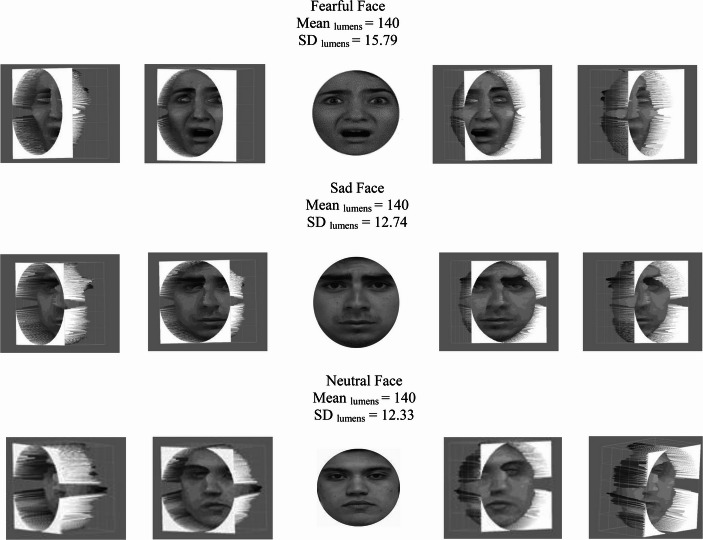
Inequivalence of contrast distribution. Three-dimensional Fourier transformations of the distribution of lumens that constitute the high components of facial images and form the recognizable structure of facial stimuli. Lines within inbox lateral presentations show the distribution of the images in lumens and, therefore, the reception of high-level characteristics from the optic nerve to the visual thalamus and the occipital cortex (Shapley, 1990; Spillmann & Werner, 2012; Wyart & Tallon-Baudry, 2009). These distribution/structural characteristics cannot be processed to equivalence between faces without disrupting the structure of emotional expressions (De Gardelle & Kouider, 2010). Stimuli with the lowest measures of dispersion per category were used in this figure to illustrate that the effect cannot be subject to diversity exceptions (see again Fig. 5)

From these techniques, grayscaling, in particular, should be examined thoroughly. For example, if we look at Fig. 3, it may be clear to the naked eye that there are luminosity differences among the processed faces. That is due to that applying grayscale corrections to these facial images maintained the colour contrast of the original images and merely transcribed it to different shades of grey. We can test this observation by applying Fourier transformations to images. Fourier transformations can be divided in continuous (CFT) and discrete (DFT) transformations, meaning, respectively, the processing of continuous stimuli, such as a short film or series of images, and the processing of a single static image. It can give us a numerical value (see Fig. 4) as well as one-dimensional and multi-dimensional outcomes for the low and high frequency components of images (see Figs. 5 and 6). The low frequency component of this methods that is most relevant to this area of research is mean luminosity. The higher components of interest are the lumens distribution, such the standard deviation of the luminosity of an image, and the three-dimensional edges and structure of the assessed image (Guo et al., 2008). We do not necessarily need to compile code for acquiring outcomes for these characteristics, there are open-source software that are available for this task (see Fig. 4; for open-source code and open-source software resources, see https://osf.io/xuvtd/).

### Empirical Illustrations, Part III: “To Unmask”

As a practical illustration, in a series of pilot studies in 2018–2019, we tested the effects of grayscale corrections in signal detection. We tested using a between-subjects design the detection performance for masked images with low, medium and high frequency lumens, and with low, medium and high-lumens pattern masks and equivalent lumens contrast using SHINE, MATLAB (Willenbockel et al., 2010).

In the first experiment, a total of 60 undergraduate psychology students (31 female) (P _(1−β)_ ≥ 0.9; *p* ≤.05; η^2^_p_ = 0.06; f = 0.25) were recruited. Their average age was 22.34 (SD = 1.91). In this experimental pilot, participants were presented with sixty fearful, sad and neutral faces, and one-hundred-eighty Gaussian blurs for 16.67 ms. These stimuli were masked with a black-and-white pattern that lasted for 116.67 ms. The experiment was divided in three sessions with 5-minute breaks between sessions and order of sessions randomised. Each session included twenty fearful, sad and neutral faces, and sixty Gaussian blurs, In one session, the faces and Gaussian blurs were adjusted at high lumens (M = 160; SD _overall average_ = 20.17), in one session, they were adjusted at medium lumens (M = 140; SD _overall average_ = 19.12) and in one session, they were adjusted at low lumens (M = 120; SD _overall average_ = 21.03). In all sessions, the stimuli were masked with an adjusted at mid-lumens values mask (M = 140; SD = 19.18). One second after each presentation in each session, participants were asked “Did you see a face (Y/N)”. After that task participants were asked to rate from “1” (not at all) to “9” (extremely) their confidence for their response.

Significant differences were shown among Sessions for Detection Performance (F (2, 118) = 372.67; *p* <.001; η^2^_p_ = 0.86; SE = 0.007; BF = +∞) and Type of Emotion (F (2, 118) = 173.89; *p* <.001; η^2^_p_ = 0.75; SE = 0.005; BF = +∞) and a significant Sessions for Detection Performance by Emotion Interaction was reported (F (4, 236) = 31.07; *p* <.001; η^2^_p_ = 0.35; SE = 0.011; BF = +∞). Further Bonferroni-corrected comparisons showed that the high-lumens session (M = 0.66; SD = 0.04) was higher for sensitivity index A compared to the low (M = 0.63; SD = 0.03; *p* <.001; d = 0.85; SE = 0.002; BF = +∞) and mid-lumens sessions (M = 0.55; SD = 0.02; *p* <.001; d = 3.48; SE = 0.001; BF = +∞). The low-lumens session was also higher than the mid-lumens session (*p* <.001; d = 3.14; SE = 0.001; BF = +∞). Similar results were reported for confidence ratings. Significant differences were shown between Sessions for Detection Performance (F (2, 118) = 1705.12; *p* <.001; η^2^_p_ = 0.97; SE = 0.004; BF = +∞) and Type of Emotion (F (2, 118) = 61.45; *p* <.001; η^2^_p_ = 0.51; SE = 0.005; BF = +∞), and a significant Session for Detection Performance by Emotion Interaction was reported (F (4, 236) = 4.35; *p* <.001; η^2^_p_ = 0.07; SE = 0.011; BF = +∞). Further Bonferroni-corrected comparisons showed that the high-lumens session (M = 8.11; SD = 0.54) was higher for confidence ratings than the low (M = 6.77; SD = 0.45; *p* <.001; d = 2.69; SE = 0.002; BF = +∞) and mid-lumens sessions (M = 4.79; SD = 0.43; *p* <.001; d = 6.8; SE = 0.001; BF = +∞). The low-lumens session was also higher than the mid-lumens session (*p* <.001; d = 4.49; SE = 0.002; BF = +∞). These results showed that the lumens level of the masked stimuli influenced detection performance and confidence for detection (see also Appendix 1A & 1B).

In the second experiment, a new sample of 60 undergraduate psychology students (33 female) (P _(1−β)_ ≥ 0.9; *p* ≤.05; η^2^_p_ = 0.06; f = 0.25) were recruited. Their average age was 21.09 (SD = 1.12). In this experiment, we manipulated the lumens contrast of the black-and-white pattern mask. Sixty fearful, sad and neutral faces, and one-hundred-eighty Gaussian blurs were presented. The stimuli were presented for 16.67 ms. They were presented in three sessions involving twenty fearful. sad and neutral faces, and sixty blurs each with a 5-minute break between each session and order of sessions randomised. All the faces were averaged for luminescence in SHINE, MATLAB (M = 140; SD _overall average_ = 23.08). In one session, the faces were masked with a high-lumens black-and-white pattern mask (M = 160; SD = 27.03), in one session, they were masked with a mid-lumens mask (M = 140; SD = 27.03), and in one session, they were masked with a low-lumens mask (M = 120; SD = 27.03). One second after each presentation in each session, participants were asked “Did you see a face? (Y/N)”. After that task participants were asked to rate from “1” (not at all) to “9” (extremely) their confidence for their response.

Significant differences were shown among Sessions for Detection Performance (F (2, 118) = 1168.73; *p* <.001; η^2^_p_ = 0.95; SE = 0.004; BF = +∞) and Type of Emotion (F (2, 118) = 16.26; *p* <.001; η^2^_p_ = 0.22; SE = 0.003; BF = +∞), and a significant Sessions for Detection Performance by Emotion Interaction was also reported (F (4, 236) = 3.21; *p* =.01; η^2^_p_ = 0.05; SE = 0.007; BF = +∞). Further Bonferroni-corrected comparisons showed that the low-lumens session (M = 0.73; SD = 0.04) was higher for sensitivity index A than the high (M = 0.71; SD = 0.03; *p* <.001; d = 0.57; SE = 0.002; BF = +∞) and mid lumens sessions (M = 0.54; SD = 0.02; *p* <.001; d = 7.45; SE = 0.001; BF = +∞). The high-lumens session was also higher than the mid-lumens session (*p* <.001; d = 5.38; SE = 0.003; BF = +∞). For confidence ratings, significant differences were shown between Sessions for Detection Performance (F (2, 118) = 2489.81; *p* <.001; η^2^_p_ = 0.97; SE = 0.005; BF = +∞), Type of Emotion (F (2, 118) = 13.75; *p* <.001; η^2^_p_ = 0.19;; SE = 0.005; BF = +∞), and a Sessions for Detection Performance by Emotion Interaction effects were revealed (F (4, 236) = 14.4; *p* <.001; η^2^_p_ =. 19; SE = 0.009; BF = +∞). Further Bonferroni-corrected comparisons showed that the low-lumens session (M = 8.03; SD = 0.59) was higher for confidence ratings than the high (M = 7.12; SD = 0.37; *p* <.001; d = 1.85; SE = 0.001; BF = +∞) and mid-lumens sessions (M = 4.79; SD = 0.36; *p* <.001; d = 6.63; SE = 0.002; BF = +∞). The high-lumens session was also higher than the mid-lumens session (*p* <.001; d = 6.38; SE = 0.001; BF = +∞). These results showed that the lumens level of the mask influenced the participants’ detection performance and confidence ratings (see also Appendix 2A & 2B).

In the third experimental pilot, a new sample of 60 undergraduate psychology students (31 female) (P _(1−β)_ ≥ 0.9; *p* ≤.05; η^2^_p_ = 0.06; f = 0.25) were recruited. Their average age was 22.21 (SD = 1.95). We repeated the designs described above after averaging in SHINE, MATLAB the masks at mid lumens (M = 140; SD = 27.03) and all the faces at mid lumens (M = 140; SD _overall average_ = 23.02). The participants engaged in three same-day sessions with a 5-minute break between sessions and including only matched for lumens among stimulus types masked faces, matched for lumens masks and, therefore, no lumens contrast between mask and masked stimuli.

In this pilot experiment, no significant differences and Bayesian evidence for equivalence of significance were revealed among Sessions for Detection Performance (F (2, 118) = 0.59; *p* =.66; η^2^_p_ < 0.01; SE = 0.007; BF = 0.31) and confidence ratings (F (2, 118) = 0.29; *p* =.75; η^2^_p_ < 0.01; SE = 0.07; BF = 0.26). No other significant results were reported for comparisons among Types of Emotion (F (2, 118) = 0.183; *p* =.165; η^2^_p_ = 0.03; SE = 0.008; BF = 1.92), and Types of Emotion and Session Order interactions for detection (F (4, 236) = 0.41; *p* =.8; η^2^_p_ < 0.01; SE = 0.12; BF = 0.13) and confidence ratings (F (4, 236) = 0.564; *p* <.001; η^2^_p_ < 0.01; SE = 0.01; BF = 0.29). Αn important outcome of this experimental pilot was that the detection performance overall across all three sessions (M = 0.511; SD = 0.977; SE = 0.012) provided Bayesian results that approximated evidence for the null (BF = 0.34). This signified that the participants responded as if they were overall marginally at-chance level detection performance for the presented faces. This result extended to sad (M = 0.505; SD = 0.989; SE = 0.013; BF = 0.05) and neutral faces (M = 0.508; SD = 0.998; SE = 0.013; BF = 0.06). It was reduced for fearful faces (M = 0.521; SD = 0.99; SE = 0.014; BF = 0.9).

In this pilot study, we came very close to providing unbiased evidence for subliminality. We were able to approximate Bayesian evidence for the null using sensitivity index A. This effect can be interpreted as an outcome of averaging mean luminosity among the presented masked faces, the presented masks, and the mask-to-masked stimuli. These manipulations could be the reason that enabled us to report a very proximate overall outcome for Bayesian evidence for the null, and substantial evidence for the null for sad and neutral faces. Our methodological framework for equivalence of contrast among all the stimuli that constituted the backward masked presentation sequence was *almost successful* (see Tsikandilakis et al., 2024; see also Fig. 5).

### The Importance of “Being Almost Successful”

In the aforementioned illustrations, we showed an application of contributing methodological resolutions relating to visual psychophysics. Despite eventually providing overall proximity to equivalence to the null, and strong evidence for the null for sad and neutral faces, we failed to achieve unbiased evidence for the unconscious presentation of fearful faces. The answer as to why this effect occurred could be related to evolutionary psychology (Öhman & Mineka, 2001; Schultz & Helmstetter, 2010; Sevenster et al., 2014; Weike et al., 2007). The perception of fearful faces could be more acute because it confers increased evolutionary survival value. Fearful expressions could signify, via an interpersonal proxy, the unseen presence of an imminent threat in our immediate environment (Öhman, 2009).

When fearful faces were presented for the same brief duration (i.e., 16.67 ms), they could have been perceived more accurately than less evolutionary important stimulus types (i.e., sad and neutral faces; see Hedger et al., 2015). This can be interpreted as a valid argument. Our research group has provided empirical evidence that could be interpreted to support this argument (Tsikandilakis et al., 2020c, 2021a, 2021b), other research groups have provided empirical evidence that could be interpreted to support this argument (Etkin et al., 2004; Hedger et al., 2015; Stein et al., 2010; Wang et al., 2017; Wieser & Keil, 2014), and, in general, it is an argument that has been widely submitted (Confer et al., 2010) and met with little resistance in psychological science (Stein et al., 2020). Nevertheless, this argument is a conceptual interpretation (see Workman & Reader, 2021). It can only be inferred as a theoretical reading from supporting empirical findings relating to the evolutionary trajectory of human emotional perception (for a comprehensive review, see Hagen, 2005).

Something else can be illustrated directly. The careful reader will have noticed that depending on the occasion, throughout this manuscript, we presented the standard deviation for luminosity either as a fixed value or an overall aggregate. We did so because in the case of pattern masks the same image was repeated and, therefore, the same standard deviation applied for lumens values. In the case of masked faces, different faces of the same and different emotional type involved differences in the distribution of luminosity (see Sarantakos, 1998). This effect occurred because the distribution of luminosity is a high-level visual component that varies for each face and contributes to the structure of the presented face and its emotional characteristics (see De Gardelle & Kouider, 2010; see Fig. 6).

We cannot change that the standard deviation of luminosity differed among stimulus types. If we manipulated our images to equivalence of lumens distribution, using MATLAB SHINE, the result would have been similar to the Gaussian blurs presented in Fig. 2, or if we endeavoured to use manual code, a series of distorted pseudo-faces, (see https://osf.io/wkhtu/; see Appendix Fig. 7). The distribution of lumens contributes to the structure of the presented face that confers emotional information (Spillmann & Werner, 2012; Wyart & Tallon-Baudry, 2009). This means that even if we achieve “Equivalence of Contrast” of the mean luminescence among faces, and among faces and masks, we cannot achieve “Equivalence of Contrast” of the distribution of luminescence among faces, and among faces and masks without changing faces to non-facial or distorted images.

Fearful faces have properties related to the distribution and structure of their facial characteristics that have been suggested to convey emotional characteristics that make them potentially more perceptible than other stimulus types under conditions of visual suppression. We do not wish to enter into a potentially circular argument on whether the higher-level characteristics of fearful faces are due to their evolutionary significance, or whether the evolutionary significance of fearful faces is a conceptual reading of their higher-level visual characteristics. We bequeath – with scepticism as to the possibility of its conceptual resolution (see Murphy & Zajonc, 1993) – this debate to dedicated reviews (see Caruana & Seymour, 2022; Schindler et al., 2021; see Fig. 6). For the purposes of the current manuscript, it is important to submit that fearful faces, as do other kinds of emotional faces, such as happy and angry faces (Tsikandilakis et al., 2020b, 2022, 2023a, b) have distinct high-level characteristics that make them more perceptible than other emotional faces, such as sad and neutral faces (Liddell et al., 2005). Inconveniently, there is very little we can do about this issue except employ our method for individual unconsciousness (Tsikandilakis et al., 2024, 2025a, b). Doing so will take us to a very different place from where we currently stand using static durations of presentation. It is, therefore, maybe more important to submit a lesson: There will not always be a “grail” answer to our experimental questions (see Oatley, 1992; see also Fig. 6). There will frequently be issues, concerns and hurdles that are pending a conclusive resolution, or may not have one within our current paradigm, and we should not forget that we ourselves are also at this point in time operating within a specific scientific paradigm that has limitations (see Pessoa & Adolphs, 2010), and we should be conscious that it does (see Fuller et al., 2013)^3^.

## The Importance of “Being Conscious”

Along these lines, throughout this manuscript, we endeavoured to present an integrative discourse of some of the things that what we should be conscious of when engaging with research relating to the unconscious. We discussed several subjects from psychological history, such as the very early beginnings of the unconscious in written language, and controversial historical episodes, to psychological science, and mathematical psychology, such as established and alternative methods of statistical analyses, signal-detection-theory metrics, cognitive and evolutionary psychological perspectives, and contemporary psychophysics and psychophysiology. If we could highlight the importance of a single and simple lesson for our readership it should be – as Heraclitus wrote 2,500 years ago – the importance of being conscious; when engaging with research into the unconscious.

Significant outcomes for unconscious processing, and their refutations are understood and influence the general public potentially now more than ever (Albarrak et al., 2021). We should already have risen to this challenge in the past years (see Amd & Passarelli, 2020; Lapate et al., 2020). In this manuscript, we discussed several resolutions towards this goal. Some were known. They are not applied (Brooks et al., 2012; Van den Bussche et al., 2009; Gambarota et al., 2022; Meneguzzo et al., 2014; Mertens & Engelhard, 2020; van der Ploeg et al., 2017). Some were novel. They should be considered and advanced (Weinberger & Stoycheva, 2019).

It is our opinion, that, in this area of psychological science, we suffer from an “inapplicability bias”: We have immense contemporary methodological resources, advances and resolutions that we do not sufficiently understand or acknowledge or appreciate and, therefore, do not apply to contemporary empirical research practice. Towards true resolutions, the interplay between awareness and implementation is very critical. Not applying resolutions to critical problems can lead to bias. Applying them without proper scholarly and methodological awareness can reduce bias but cannot lead to scientific progress. Applying them with proper scholarly and methodological awareness is the foundation for constructively advancing, understanding, engaging, undertaking and communicating research into the unconscious. This ought to be our *moral imperative*, if we truly want to be, or become, *conscious* readers, students, scholars and researchers of the *unconscious* (Bargh, 2017; Bornstein, 1989; Erdelyi, 2004; Pessoa & Adolphs, 2010; Theus, 1994; Vadillo et al., 2020; Wyer & Scull, 2014).

## Conclusions

In this manuscript, we discussed several perspectives of the unconscious. We discussed perspectives ranging from ancient philosophy to contemporary psychophysics. We emphasized how formative historical episodes, and early theoretical models clashed with empirical perspectives. We showed how this clash and eventual concerted fermentation of ideas created the methodological paradigm we use today. We showed that we have forgotten many of these formative instances. Conversely, we showed that, as we have come to forget how conjectural theory and applied empiricism contributed to methodological progress, we have forgotten our *moral imperative* to apply methodological progress to empirical practice. We meta-analytically showed that we are not using, applying and employing our methodological advances to empirical research. We showed that we are at this point in time – despite all we have achieved – repeating the errors of the past; charging forth, almost blindly and unconsciously, to *the doing* of research into the unconscious. We argued that following this path will lead to irreparable schisms and crises in psychological science. We invited the reader to appreciate how applying the methodological advances, and advancing the methodological applications in this area are inviolate responsibilities for being *conscious* readers, students, scholars and researchers of the *unconscious*.

## Figures and Tables

**Fig. 7:**
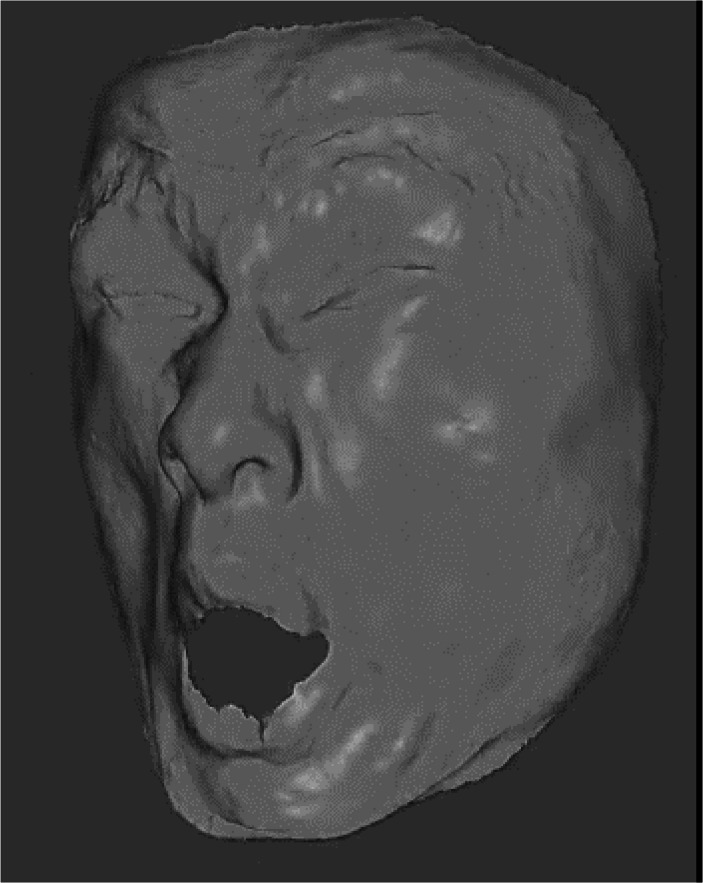
Manual code for the equivalence of the SD for lumens among stimulus types. Source: https://osf.io/wkhtu/

## Data Availability

The materials for the current work have been made available at (https://osf.io/wkhtu/)(https://osf.io/wkhtu/).

## Appendix 1

### 1A: Type of Emotion Bonferroni Corrected Comparisons, Pilot One

Detection Performance

**Table:**

Estimates				
Emotion	Mean	Std. Error	95% Confidence Interval	
			Lower Bound	Upper Bound
Fear	,659	,003	,652	,666
Sadness	,596	,003	,590	,601
Neutral;	,595	,003	,590	,600

**Table:**

Pairwise Comparisons						
(I) Emotion	(J) Emotion	Mean Difference (I-J)	Std. Error	Sig.^b^	95% Confidence Interval for Difference ^b^	
					Lower Bound	Upper Bound
Fear	Sadness	,063^*^	,004	,000	,054	,073
	Neutral	,064^*^	,004	,000	,054	,074
Sadness	Fear	-,063^*^	,004	,000	-,073	-,054
	Neutral	,001	,004	1,000	-,008	,010
Neutral	Fear	-,064^*^	,004	,000	-,074	-,054
	Sadness	-,001	,004	1,000	-,010	,008

Based on estimated marginal means

*The mean difference is significant at the,05 level.

b. Adjustment for multiple comparisons: Bonferroni.

**Table:**

Estimates				
Measure: Confidence				
Emotion	Mean	Std. Error	95% Confidence Interval	
			Lower Bound	Upper Bound
Fear	6,860	,032	6,795	6,925
Sadness	6,392	,039	6,315	6,469
Neutral	6,422	,033	6,357	6,487

**Table:**

Pairwise Comparisons						
Measure: Confidence						
(I) Emotion	(J) Emotion	Mean Difference (I-J)	Std. Error	Sig.^b^	95% Confidence Interval for Difference ^b^	
					Lower Bound	Upper Bound
Fear	Sadness	,468^*^	,047	,000	,351	,585
	Neutral	,438^*^	,045	,000	,328	,548
Sadness	Fear	-,468^*^	,047	,000	-,585	-,351
	Neutral	-,030	,050	1,000	-,152	,092
Neutral	Fear	-,438^*^	,045	,000	-,548	-,328
	Sadness	,030	,050	1,000	-,092	,152

Based on estimated marginal means

*The mean difference is significant at the,05 level.

b. Adjustment for multiple comparisons: Bonferroni.

### 1B: Type of Emotion to Session Type Bonferroni Corrected Comparisons, Pilot One

**Table:**

4. Session * Emotion					
Measure: Detection					
Session	Emotion	Mean	Std. Error	95% Confidence Interval	
				Lower Bound	Upper Bound
1	1	,728	,005	,718	,739
	2	,635	,006	,623	,648
	3	,630	,005	,620	,640
2	1	,690	,007	,677	,703
	2	,605	,004	,596	,613
	3	,606	,004	,598	,614
3	1	,559	,005	,549	,568
	2	,547	,004	,540	,554
	3	,549	,004	,542	,556

## Appendix 2

### 2A: Type of Emotion Bonferroni Corrected Comparisons, Pilot Two

**Table:**

Estimates				
Measure: Detection				
Emotion	Mean	Std. Error	95% Confidence Interval	
			Lower Bound	Upper Bound
Fear	,674	,003	,669	,679
Sadness	,653	,003	,646	,660
Neutral	,652	,003	,646	,659

**Table:**

Pairwise Comparisons						
Measure: Detection						
(I) Emotion	(J) Emotion	Mean Difference (I-J)	Std. Error	Sig.^b^	95% Confidence Interval for Difference ^b^	
					Lower Bound	Upper Bound
Fear	Sadness	,021^*^	,004	,000	,012	,030
	Neutral	,022^*^	,004	,000	,011	,033
Sadness	Fear	-,021^*^	,004	,000	-,030	-,012
	Neutral	,001	,005	1,000	-,011	,012
Neutral	Fear	-,022^*^	,004	,000	-,033	-,011
	Sadness	-,001	,005	1,000	-,012	,011

Based on estimated marginal means

*The mean difference is significant at the,05 level.

b. Adjustment for multiple comparisons: Bonferroni.

### 2B: Type of Emotion to Session Type

**Table:**

4. Session * Emotion					
Measure: Confidence					
Session	Emotion	Mean	Std. Error	95% Confidence Interval	
				Lower Bound	Upper Bound
1	1	7,185	,079	7,028	7,342
	2	7,159	,076	7,007	7,311
	3	7,192	,075	7,042	7,342
2	1	4,818	,044	4,729	4,907
	2	4,768	,049	4,669	4,866
	3	4,796	,045	4,706	4,885
3	1	8,468	,035	8,399	8,538
	2	7,804	,052	7,701	7,907
	3	7,809	,057	7,696	7,923

Bonferroni Corrected Comparisons, Pilot Two

**Table:**

Estimates				
Measure: Detection				
Emotion	Mean	Std. Error	95% Confidence Interval	
			Lower Bound	Upper Bound
Fear	6,824	,032	6,761	6,887
Sadness	6,577	,036	6,504	6,650
Neutral	6,599	,039	6,520	6,678

**Table:**

Pairwise Comparisons						
Measure: Detection						
(I) Emotion	(J) Emotion	Mean Difference (I-J)	Std. Error	Sig.^b^	95% Confidence Interval for Difference ^b^	
					Lower Bound	Upper Bound
Fear	Sadness	,247^*^	,048	,000	,129	,365
	Neutral	,225^*^	,054	,000	,092	,357
Sadness	Fear	-,247^*^	,048	,000	-,365	-,129
	Neutral	-,022	,054	1,000	-,156	,111
Neutral	Sadness	-,225^*^	,054	,000	-,357	-,092
	Neutral	,022	,054	1,000	-,111	,156

Based on estimated marginal means

**Table:**

4. Session * Emotion					
Measure: Confidence					
Session	Emotion	Mean	Std. Error	95% Confidence Interval	
				Lower Bound	Upper Bound
1	1	,723	,006	,711	,734
	2	,699	,008	,683	,715
	3	,701	,008	,686	,717
2	1	,544	,002	,540	,547
	2	,537	,002	,534	,541
	3	,540	,002	,536	,544
3	1	,755	,005	,746	,764
	2	,723	,006	,711	,735
	3	,716	,005	,706	,725

## Footnotes

1 It should be noted that several other factors in neutral masks, such as age, gender, cultural and racial unfamiliarity, racial incongruency and also state-emotional characteristics (Lee et al., 2008) can further influence this effect (Kim et al., 2010; see also Adams et al., 2016).

2 To help the interested researcher explore and potentially implement these changes in their own research, we have made open access a set of pattern masks that replicated the above outcomes and accompanying easy-to-use coding scripts for including these masks to their experimental designs (see https://osf.io/wkhtu/).

3 The coding scripts (including tutorial information) for this section, including DFT and three-dimensional Fourier transformations scripts (https://osf.io/xuvtd/) as well as the coding script for averaging mean luminosity between stimuli (https://osf.io/5zp3r/), have been made available by our group in open-source format (https://osf.io/wkhtu).
